# Metabolic and Environmental Benefits of Following the Healthy and Sustainable Dietary Recommendations for the Spanish Population: The AWHS Study

**DOI:** 10.3390/nu17233725

**Published:** 2025-11-27

**Authors:** Sofía Gimeno-Ruiz, Pilar Guallar-Castillón, Esther López-García, Carolina Torrijo-Belanche, Ainara Muñoz-Cabrejas, María Morales-Suárez-Varela, Belén Moreno-Franco

**Affiliations:** 1 Department of Preventive Medicine and Public Health, Universidad de Zaragoza, 50009 Zaragoza, Spain; gimenoruizsofia@gmail.com (S.G.-R.); carolinatorrijob@gmail.com (C.T.-B.); mbmoreno@unizar.es (B.M.-F.); 2 Instituto Madrileño de Estudios Avanzados en Alimentación (IMDEA-Food Institute), Campus de Excelencia Internacional Universidad Autónoma de Madrid + Consejo Superior de Investigaciones Científicas (CEI UAM + CSIC), 28049 Madrid, Spain; esther.lopez@uam.es; 3 Department of Preventive Medicine and Public Health, School of Medicine, Universidad Autónoma de Madrid, 28029 Madrid, Spain; 4 Department of Epidemiology, New York University School of Global Public Health, New York, NY 10003, USA; 5 Instituto de Investigación Sanitaria Aragón, Hospital Universitario Miguel Servet, 50009 Zaragoza, Spain; ainaramunozc@gmail.com; 6 Department of Preventive Medicine and Public Health, Food Sciences, Toxicology and Forensic Medicine, Universitat de València, 46100 Valencia, Spain; maria.m.morales@uv.es; 7 Centro de Investigación Biomédica en Red de Epidemiología y Salud Pública (CIBERESP), 28029 Madrid, Spain; 8 Centro de Investigación Biomédica en Red de Enfermedades Cardiovasculares (CIBERCV), 28029 Madrid, Spain

**Keywords:** dietary guidelines, sustainability, epidemiology, metabolic syndrome

## Abstract

**Background/Objectives:** Emerging approaches aim to protect both human and environmental health. Therefore, we hypothesise that adherence to the Healthy and Sustainable Dietary Recommendations for the Spanish Population Index (HS-DRSI) is inversely associated with the occurrence of Metabolic Syndrome (MetS) and promotes environmental sustainability. **Methods**: A cross-sectional analysis was performed with 2286 middle-aged men, with no previous cardiovascular disease and belonging to the Aragon Workers’ Health Study (AWHS). Diet was collected with a 136-item food-frequency questionnaire. Adherence to the recommendation was assessed with the HS-DRSI, which assigns one point for each of 19 food categories. The National Cholesterol Education Programme-Adult Treatment Panel III (NCEP-ATP III) definition was used to define MetS. Logistic regressions were used to estimate the association between adherence to the HS-DRSI and presence of MetS. Environmental assessment was calculated based on the 2016 European database. **Results**: Higher adherence to the HS-DRSI was inversely associated with MetS and elevated waist circumference. The odds ratio (OR) of having MetS for participants in the highest (8–13 points) vs. lowest (1–5 points) quartile of adherence to the diet score was 0.72 (95% CI, 0.52–0.99) and the OR of elevated waist circumference was 0.69 (95% CI, 0.48–0.99), with consistent results per 1 SD increase (2 points). In addition, greenhouse gas emissions (GHGE) decreased progressively across adherence quartiles (from 6.10 to 5.70 kg CO_2_-eq/day). **Conclusions**: Adherence to the HS-DRSI was associated with a lower risk of MetS and may contribute to lower GHGE production.

## 1. Introduction

Non-communicable diseases have become a major global health burden, with metabolic syndrome (MetS) emerging as a widespread epidemic [1,2]. This syndrome includes several vascular risk factors: high waist circumference, high plasma glucose, high blood pressure, and atherogenic dyslipidemia [3]. If untreated, MetS is significantly associated with an increased risk of developing type 2 diabetes and atherogenic cardiovascular disease (CVD) [4]. Therefore, MetS can even help prevent subclinical CVD by identifying individuals at high risk of complications [5]. Recent evidence suggests that the most effective strategy for managing MetS involves a combination of reduced caloric intake patterns, such as the Dietary Approaches to Stop Hypertension (DASH) diet and the Mediterranean diet, together with aerobic exercise [6,7]. In recent years, these dietary patterns have also been evaluated not only for their health benefits but also for their environmental impact [8], including aspects of food production, processing, distribution, and consumption [9]. This aligns with the Sustainable Development Goals of the 2030 Agenda, which emphasize that the food chain should promote social, economic and environmental sustainability [10]. Similarly, the One Health approach recognizes the complex relationships between food systems and the natural world in which they are embedded [11]. These perspectives are progressively shaping governmental recommendations and public health policies.

In this context, the Scientific Committee of the Spanish Agency for Food Safety and Nutrition (AESAN) updated the national dietary guidelines in 2022, integrating sustainability criteria [12]. The updated recommendations for the Spanish population proposed an increment of daily consumption of plant products such as fruits, vegetables and legumes, preference for whole grains and healthy fats, and promotion of tap water whenever possible. They also advised reducing processed meats, saturated fats, sugars, and salt, while encouraging local food choices, minimizing plastic packaging, and reducing food waste [12].

To assess compliance with this guideline, the Healthy and Sustainable Dietary Recommendations for the Spanish Population Index (HS-DRSI) was developed in 2025 [13]. When applied to a representative Spanish cohort, higher adherence to the HS-DRSI was associated with a lower risk of all-cause mortality [13]. This association may be explained by the fact that the HS-DRSI reflects the overall dietary pattern and was developed based on the eating habits of the Spanish population, including commonly consumed and culturally integrated foods. Although food components [14] and dietary patterns [15,16,17,18] have been studied for the prevention or management of the MetS, the association between a sustainable dietary approach and the MetS remains unknown.

Therefore, we hypothesise that HS-DRSI is inversely associated with the occurrence of MetS and promotes environmental sustainability in a well-characterised sample of working male adults.

## 2. Materials and Methods

### 2.1. Study Population

We carried out a cross-sectional study with a subsample from the Aragon Workers’ Health Study (AWHS), whose design and methodology have been previously described [19]. Briefly, the AWHS is a longitudinal cohort based on annual physical examinations of 5678 workers from an automobile assembly plant in Spain. Participants were recruited during a standardized clinical examination in 2009–2010 (participation rate 95.6%) with the aim of assessing the CVD risk factors and their association with metabolic abnormalities and subclinical atherosclerosis. Between 2011 and 2014, participants, aged 39 to 59 years and free from CVD at baseline, attended extended examinations that included interviews with questionnaires on diet and lifestyle. Of the 2667 participants who attended the examinations, we excluded women due to their small number (n = 137), participants with extreme total energy intake (<600 or >4200 kcal; n = 139), and those with missing data on diet or MetS components (n = 105), so the final sample comprised 2286 men. The study was conducted according to the guidelines of the Declaration of Helsinki and was approved by the clinical research ethics committee of Aragon (CEICA) (PI07/09; 16 May 2007). All participants provided written informed consent.

### 2.2. Data Collection

#### 2.2.1. Diet Assessment and the Healthy and Sustainable Dietary Recommendations for the Spanish Population Index

The habitual diet during the year prior to the interview was assessed using a 136-item food frequency questionnaire (FFQ), previously validated in Spain [20]. The FFQ grouped foods into the following categories: dairy products, eggs, meat and fish, vegetables, fruit, legumes and cereals, oils and fats, pastries and cakes, miscellaneous and beverages. For each food included in the questionnaire, serving size was specified with the choice between nine frequencies of consumption from “never or almost never” to “more than six times a day”. We used the collected information to calculate the consumption of each of the HS-DRSI components [13], except water, which is not recorded in the FFQ (Table 1). Finally, participants were divided into HS-DRSI quartiles based on their scores: 5 or fewer points (first quartile), 6 points (second quartile), 7 points (third quartile), and 8 or more points (fourth quartile).

#### 2.2.2. Criteria for Diagnosing Metabolic Syndrome

The modified National Cholesterol Education Program-Adult Treatment Panel III (NCEP-ATP III) definition [3] was used to diagnose MetS if participants met at least 3 of the following 5 criteria: abdominal obesity, defined as having a waist circumference of ≥102 cm; fasting glucose of ≥100 mg/dL, or use of drug treatment for elevated glucose; systolic blood pressure of ≥130 mmHg and/or diastolic blood pressure of ≥85 mm Hg, or use of antihypertensive medication; serum triglycerides levels of ≥150 mg/dL, or use of drug treatment for hypertriglyceridemia; and serum high-density lipoprotein cholesterol (HDL-c) of <40 mg/dL.

#### 2.2.3. Baseline Information on Covariates

Sociodemographic (age, sex, and work shift), clinical, and laboratory data was collected during the annual medical examination. The physical examination included height, weight, waist circumference, and arterial blood pressure. Waist circumference was obtained using a flexible, non-extendable measuring tape (Gulick model 67109), which was verified and revised monthly versus another tape that was calibrated every three years. Blood pressure was recorded after a 5-min rest period, using the average of consecutive automatic readings from a digital sphygmomanometer (OMRON M10-IT. Healthcare Co., Ltd., Kyoto, Japan). Medical history and current medication were also documented.

Fasting blood samples (>8 h) were analyzed for serum glucose, serum triglycerides, and total HDL-c using a spectrophotometer (Chemical Analyzer ILAB 650, Instrumentation Laboratory, Bedford, MA, USA). Low-density lipoprotein cholesterol (LDL-c) was calculated using the Friedewald equation when the triglyceride level was below 400 mg/dL. Hypertension was defined as systolic blood pressure ≥ 140 mm Hg, or diastolic blood pressure ≥ 90 mm Hg, or use of antihypertensive medication [21]. Diabetes was defined as fasting plasma ≥ 126 mg/dL or reported use of hypoglycemic medication [21]. Dyslipidemia was defined as total cholesterol ≥ 240 mg/dL, or LDL-c ≥ 160 mg/dL, or HDL-c < 40 mg/dL, or use of lipid-lowering medication [22].

Smoking status was obtained through an interview. Participants who had smoked at least 50 cigarettes in their lifetime or reported having smoked within the past year were classified as ever-smokers. Those who did not meet these criteria were categorized as never-smokers.

Physical activity was assessed using the validated Spanish version of the engagement in physical activity used in the Nurses’ Health Study and the Health Professionals’ Follow-up Study [23]. Each activity was assigned to a metabolic value from Ainsworth’s compendium [24], multiplied by its reported frequency, and summed to obtain weekly metabolic equivalents-h (METs).

#### 2.2.4. Environmental Assessment of Greenhouse Gas Emissions

The sustainability of the diet was assessed using the 2016 European database [25], which reports greenhouse gas emissions (GHGE) in kg CO_2_-equivalents per kg of food. Based on life cycle assessments, this database includes emissions from agricultural production, processing, cooking, storage, and packaging, but excludes transportation and consumer travel. For each participant, daily food intake reported through the FFQ (in kg/day) was multiplied by the corresponding GHGE. The sum of all food items yielded the total daily dietary emissions per participant. The averages of these values were then grouped according to HS-DRSI quartiles.

### 2.3. Statistical Analysis

Analysis was performed using Stata statistical software (version 15). The associations between the quartiles of the HS-DRSI and MetS were examined using logistic regression models and adjusted for covariates. Additionally, we examined the association of a 1-standard deviation (SD) increment in the HS-DRSI with MetS. The models were initially adjusted for age, and then for the following additional factors: work shift, body mass index (BMI) derived from the weight and height of a person in kg/m^2^, smoking status, physical activity, total energy intake, and alcohol intake. The same analyses were performed for MetS criteria independently. Odds ratios (ORs) and their 95% confidence intervals (CIs) were calculated, with a *p* value below 0.05 being considered statistically significant. Finally, the mean and its 95% CI for total GHGE emissions were calculated for each adherence quartile.

## 3. Results

### 3.1. Baseline Characteristics of Participants

The sample included 2286 men with a mean age of 50.9 years (3.9 SD). Compared with those in the lowest quartile (Q1; 1–5 pts) of HS-DRSI adherence, those in the highest quartile (Q4; 8–13 pts) had higher levels of physical activity, higher HDL-c concentrations, lower triglyceride concentrations, and a higher prevalence of hypertension and dyslipidemia (Table 2).

The median score (50th percentile) for the HS-DRSI fell between 5 and 6 points out of a possible 19 (Table S1). Certain components of the HS-DRSI had particularly low levels of compliance. Specifically, consuming 4 servings of legumes per day, limiting meat intake to fewer than 3 servings per week, consuming processed meat less than once per week, and limiting overall processed food intake to less than once per week were each adhered to by fewer than 1.0% of participants. Conversely, the components to which more participants adhered (about 86.0%) were limiting eggs to fewer than 4 per week and dairy to fewer than 3 per day (Table S2). The median of potato intake was 95.71 g per day with a moderate adherence (63.8%).

Differences in dietary intake were found among quartiles of the HS-DRSI. Those with the highest adherence consumed less total energy, carbohydrates, protein, and fat, but more fibre. Notably, legumes were the only food category to remain constant across quartiles (Table S3).

### 3.2. Associations Between the HS-DRSI and MetS and Its Components

A total of 632 cases of MetS were identified, representing a prevalence of 27.6%. The respective prevalences for the diagnosis criteria were 32.4% for elevated waist circumference, 38.2% for elevated fasting glucose, 57.0% for elevated blood pressure, 38.0% for elevated serum triglycerides, and 9.4% for reduced HDL-c.

**Figure 1:**
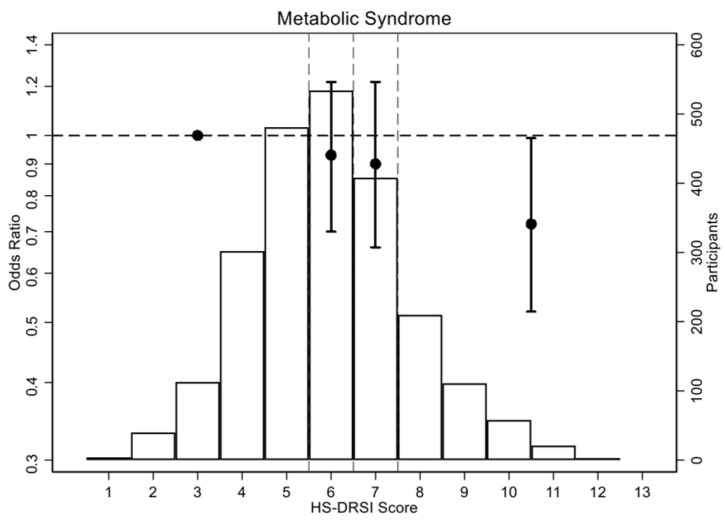
Odds ratios (OR) (95% CI) for metabolic syndrome according to the healthy and sustainable dietary recommendations for Spanish population index quartiles. The circles show the ORs, with the vertical lines representing the 95% CIs. The horizontal dashed line indicates the reference point (OR = 1). The vertical dashed lines divide the HS-DRSI score quartiles (Q1: 1–5; Q2: 6; Q3: 7; Q4: 8–13). The histogram illustrates the distribution of HS-DRSI scores.

We found an inverse association between adherence to the HS-DRSI and the occurrence of MetS. The prevalence of MetS was 24.0% among participants with a high level of adherence, compared with 27.7% among those with the lowest level. After multivariable adjustment, OR for participants in the Q4 was 0.72 (95% CI, 0.52–0.99) compared to those in the Q1. Each 1 SD increase in HS-DRSI was associated with an OR for MetS of 0.86 (95% CI, 0.77–0.97), with a statistically significant linear trend (*p* = 0.013) (Table 3; Figure 1 and Figure S1).

**Figure 2:**
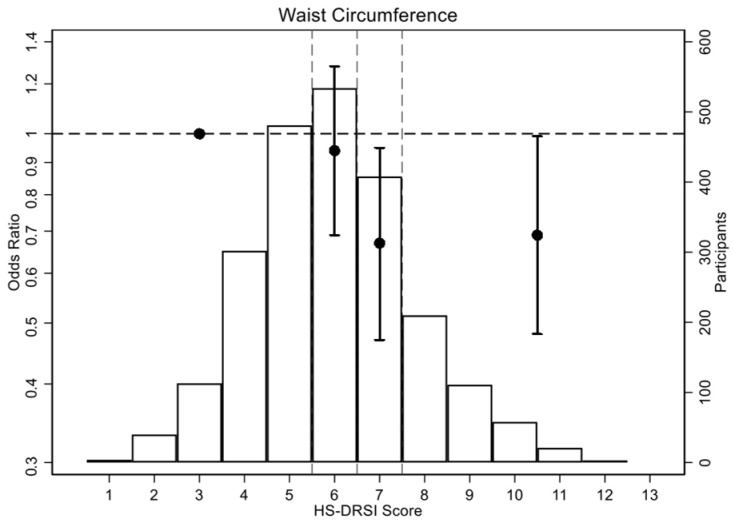
Odds ratios (OR) (95% CI) for waist circumference criteria according to the healthy and sustainable dietary recommendations for Spanish population index quartiles. The circles show the ORs, with the vertical lines representing the 95% CIs. The horizontal dashed line indicates the reference point (OR = 1). The vertical dashed lines divide the HS-DRSI score quartiles (Q1: 1–5; Q2: 6; Q3: 7; Q4: 8–13). The histogram illustrates the distribution of HS-DRSI scores.

For abdominal obesity, participants in Q4 showed an OR of 0.69 (95% CI, 0.48–0.99), compared with Q1. The OR per 1 SD increase in HS-DRSI was 0.80 (95% CI, 0.70–0.92), with a *p*-value of 0.001. For elevated triglycerides, a significant linear trend was observed (*p* = 0.017), with an OR per 1 SD increase of 0.90 (95% CI, 0.83–0.98) when adjusted for age, but this lost significance when adjusting for multivariable factors. No significant associations were found for the criteria of high fasting glucose, blood pressure, triglyceride or low HDL cholesterol (Table 3; Figure 2 and Figure S1).

### 3.3. Link Between the HS-DRSI and GHGE Production

Regarding the environmental assessment, the mean GHGE for all participants was 5.97 kg CO_2_-eq/day. Red meat consumption contributed 43.1% of total emissions, increasing to 51.0% when white meat was also included. When stratified by quartiles of adherence to HS-DRSI, the average GHGE was 6.10 (95% IC, 6.00–6.20), 6.00 (95% IC, 5.86–6.14), 5.88 (95% IC, 5.72–6.04), and 5.70 (95% IC, 5.54–5.86) kg CO_2_-eq/day for Q1 to Q4, respectively (Table S4).

## 4. Discussion

In this relatively large and well-characterised population, adherence to the HS-DRSI was associated with lower odds of MetS and resulted in lower GHGE production. This association was primarily driven by the abdominal obesity component. These findings support that integrating sustainability criteria into dietary guidelines may simultaneously promote cardiometabolic health from early stages and reduce environmental impact.

### 4.1. Participants’ Adherence to the HS-DRSI

In our study, only 17.7% of participants had an HS-DRSI adherence of 8 or higher out of 19, and only one person achieved a maximum score of 13 (Table S1). This indicates very low adherence to the index, since even those in the highest category were in the middle of the scoring range. Ideally, higher adherence (about 15–19 points) would be expected to meet the recommendations of the HS-DRSI, which could have important implications for metabolic and environmental health. Adherence to the HS-DRSI was slightly higher in the ENRICA cohort study [13], which is a representative sample of Spanish adults. However, it is noteworthy that our participants reported a higher intake of vegetables, fruits, and especially fish and seafood. These findings from both the AWHS and ENRICA cohorts highlight that, although Spain has traditionally been linked to Mediterranean dietary patterns, current adherence to healthy diets is moderate [26].

Enhancing adherence among AWHS participants remains necessary. As shown in our results, an increase of 1 SD (2 points) in adherence would be an adequate and achievable goal for improving metabolic health. In addition to increasing the consumption of vegetables, whole grains, and oily fish, attention should also be given to improving the intake of components that currently receive the lowest scores. This involves reducing meat intake, particularly processed meat, and increasing legume consumption, as the HS-DRSI emphasises plant-based protein over meat. The overconsumption of processed meat may be partly explained by the common practice of eating deli sandwiches in this population, especially among workers [27]. Moreover, this index is very restrictive when it comes to processed products, as it does not allow for even one serving per week. Similarly, although the average intake of legumes was around once or twice per week, which is acceptable in other Mediterranean indices [28], it falls short for the HS-DRSI. A similar issue arises with olive oil, because although preference over other vegetable oils and butter was observed, the HS-DRSI requirement includes olive oil as the only source of fat. Conversely, eggs and dairy products are consumed in moderation, which aligns perfectly with the requirements of this index.

The most notable update to the 2022 Spanish dietary guidelines [12] is the inclusion of sustainability requirements, which shift the focus beyond just nutritional needs. Similarly, the 2019 EAT-Lancet Commission introduced the Planetary Health Diet (PHD), which also addresses both human and planetary health [29]. Although other dietary guidelines may not explicitly address sustainability, many of their recommendations overlap, reflecting the interconnection between nutritional quality and environmental impact [30]. For example, the Mediterranean, Nordic, and DASH diets also promote the intake of fruits, vegetables, whole grains, white meat, oily fish, and minimally processed food products [31]. For this reason, the HS-DRSI emphasizes again promoting healthier dietary choices, such as buying whole-grain instead of white bread and informing people about added salt to reduce its consumption.

### 4.2. Associations Between the HS-DRSI and MetS and Its Components

In our study, we observed a 28% reduction in the occurrence of MetS when comparing extreme quartiles. Our results are consistent with the 26% reduction reported in a cross-sectional analysis examining adherence to the PHD [32]. Both studies showed similar risk estimation, although our study used an index specifically developed for the Spanish population, while the other applied the global PHD index to an Iranian cohort with distinct dietary habits (e.g., lower red meat and processed food intake, and higher fruit and vegetable consumption) [32]. Similarly, a recent study of 100,013 Chinese adults with a 5-year follow-up reported a 28% lower risk of developing MetS when comparing extreme adherence categories to a plant-based diet [33]. All these patterns appear to reduce the risk of MetS, despite using different definitions of MetS and not always specifically addressing environmental health.

Focusing on MetS components, our results showed a reduced risk of abdominal obesity with increased adherence to the index. In addition, we observed a lower risk of elevated triglycerides and a trend toward higher HDL-c levels, though these were not statistically significant. Previous studies on the PHD [32] and plant-based [33] diet have also reported a reduced risk of abdominal obesity, probably due to the calorie control required. Our results are also consistent with the findings regarding the association between the Mediterranean diet and MetS. A meta-analysis of 41 observational studies published up to 2021 found that high adherence to the Mediterranean diet was associated with improvements across all 5 components of MetS. However, statistically significant results were only for waist circumference, triglycerides and HDL-c criteria [34].

Previous studies have investigated the associations of individual foods with metabolic health, which could provide insight into the mechanisms underlying the relationship between HS-DRSI and MetS. For example, a study also performed in the AWHS found that consuming low-quality carbohydrates was linked to up to 48% higher risk of MetS, with triglyceridemia being the main contributor [35]. Moreover, an umbrella review of 17 systematic reviews and meta-analyses conducted between 2014 and 2021 showed that high intake of nuts, fish, dairy products, and white meat was associated with a lower risk of MetS in both high-vs-low and per-serving comparisons. In contrast, high intake of processed and red meat was linked to a higher risk of MetS. For other food groups, such as vegetables, fruit, whole grains, legumes, eggs, and sweetened beverages, the results were either inconsistent or not statistically significant. However, the general trend supported the idea that plant-based foods are protective and sugary drinks are harmful [36]. Among these results, the high HS-DRSI requirement for legume consumption is notable, given the lack of a statistically significant association. Similarly, a cross-sectional study of 16,358 participants from the NutriNet-Santé cohort conducted in 2025 showed that the highest legume consumption (>75 g/d) was not related to a lower prevalence of cardiometabolic parameters compared to no consumption [37]. This is probably due to the generally low consumption of this food or the limited variability in intake among participants. Nevertheless, this study has clear social relevance because legumes are affordable and convenient, especially when sold pre-cooked and without added salt, and their versatility allows inclusion in both hot dishes and salads, which highlight their role in public health recommendations.

Adhering to this dietary pattern entails the regular consumption of key components that collectively support cardiovascular health. For example, dietary fibre enhances glycemic control, promotes satiety and modulates gut microbiota, thereby reducing visceral adiposity and improving insulin sensitivity [38]. Monounsaturated fatty acids, primarily derived from olive oil along with its naturally occurring polyphenols, also play a crucial role by improving lipid profiles and reducing oxidative stress [39]. Furthermore, antioxidants such as vitamins A, C and E protect against cellular damage, while essential micronutrients (including magnesium, zinc, selenium, iron, calcium and B vitamins) are fundamental to metabolic regulation, immune function and vascular health [40]. However, given the complexity of MetS, a combination of a healthy diet and regular physical activity is needed to manage it effectively [41,42].

### 4.3. Link Between the HS-DRSI and GHGE Production

Within our participants, the overall low adherence to the index meant that even those in the Q4 showed average GHGE of 5.70 (95% IC, 5.54–5.86) kg CO_2_-eq/day, exceeding the current South European average of 4.90 kg CO_2_-eq/day [43]. A higher adherence to the HS-DRSI requires increasing the consumption of fruits, vegetables, nuts and legumes which have been shown to be more sustainable and accessible [44,45,46]. For example, a school-canteen intervention that substituted most meat consumption for legumes significantly reduced the carbon and water footprints [47]. However, eliminating animal-sourced foods is not a realistic goal in all situations, and replacing them with plant-origin alternatives could have a worse environmental impact, depending on the production context [48]. Therefore, the HS-DRSI dietary pattern should be examined in greater detail as a sustainable option, since it promotes traditional, seasonal, and minimally processed foods, along with a balanced combination of animal and plant-based products.

### 4.4. Strengths and Limitations

Our study has several strengths. It included a relatively large cohort and used validated measurements for dietary intake and MetS components. In addition, we used a novel dietary index that incorporates environmental sustainability and reflects the complexity of overall dietary patterns. However, some limitations should be noted. First, a cross-sectional design limits the establishment of causal relationships or determines the temporality of associations. Second, the use of middle-aged men restricts the generalizability of our findings to other populations. Finally, despite adjusting for multiple potential confounders, some residual confounding may remain. Therefore, future research should include longitudinal studies of different populations to clarify the relationship between the HS-DRSI and metabolic health. The environmental assessment should also be expanded to include a more detailed investigation of GHGE production and other indicators, such as land use.

## 5. Conclusions

In middle-aged men, adherence to the healthy and sustainable dietary recommendations for the Spanish population was associated with a lower risk of MetS and may contribute to lower GHGE production. Adherence to the index was also shown to reduce the risk of elevated waist circumference. These findings suggest that public health policies based on these recommendations could simultaneously promote cardiovascular health from early stages and contribute to environmental protection. At present, the HS-DRSI may serve as a holistic strategy to address complex health challenges.

## Figures and Tables

**Table 1:** The components and dichotomous punctuation of the healthy and sustainable dietary recommendations for Spanish population index.

	Index Components ^1^	0 Points	1 Points
1	Vegetables and fruits, excluding fruit juice	<5 servings/d	≥5 servings/d
2	At least 3 servings of V&F are vegetables	No	Yes
3	Potatoes and other tubers	≥Median	<Median
4	Cereals, depending on the level of energy requirement ^2^	Lower: >4 servings/d	Lower: ≤4 servings/d
		Medium: >5 servings/d	Medium: ≤5 servings/d
		Higher: >6 servings/d	Higher: ≤6 servings/d
5	At least half of the servings/d of cereals are whole grain products	No	Yes
6	Legumes	<4 servings/wk	≥4 servings/wk
7	Nuts	<3 servings/wk	≥3 servings/wk
8	At least 2 servings of plant-based protein foods (nuts and legumes) per day ^3^	No	Yes
9	Fish and seafood	<3 servings/wk	≥3 servings/wk
10	At least half of the servings of fish and seafood are oily fish	No	Yes
11	Eggs	>4 eggs/wk	≤4 eggs/wk
12	Dairy	>3 servings/d	≤3 servings/d
13	All types of meat	>3 servings/wk	≤3 servings/wk
14	At least half of the servings of meat are white meat from poultry or rabbit	No	Yes
15	Olive oil for cooking and food dressing	No	Yes
16	Minimize consumption of processed meat ^4^	≥1 serving/wk	<1 serving/wk
17	Minimize consumption of other processed foods high in sugars, fats, and salt (e.g., industrially baked foods, cookies, chocolate, sweets, snacks)	≥1 serving/wk	<1 serving/wk
18	Minimize consumption of salt	No	Yes
19	Minimize consumption of sweetened beverages (sugar-sweetened, artificially sweetened, fruit juices) and energy drinks	≥1 serving/wk	<1 serving/wk
20	Water as the drink of choice	No	Yes

^1^ 1 serving of vegetables = 175 g; 1 serving of fruits = 160 g; 1 serving of cereals = 50 g of bread or 70 g of pasta/rice; 1 serving of legumes = 55 g dried or 170 g cooked; 1 serving of nuts = 25 g; 1 serving of fish and seafood = 137.5 g; 1 serving of eggs = 1 egg (~58 g); 1 serving of dairy = 225 mL of milk, 105 g of fresh cheese, 50 g of hard cheese or 125 g yogurt and other fermented milks; 1 serving of meat = 112.5 g; 1 serving of baked food: 70 g; 1 serving of cookies = 30 g; 1 serving of chocolate = 30 g; 1 serving of sweets = 10 g; 1 serving of snacks = 30 g; 1 serving of jam = 20 g; 1 serving of breakfast cereals = 40 g; 1 servings of sauces = 20 g; 1 serving of processed soups = 200 g; 1 serving of “croqueta” = 150 g; 1 serving of pizza = 150 g and 1 serving of sweetened or energy beverages = 200 mL. ^2^ Energy requirements vary based on the level of physical activity and are categorized as follows: sedentary (inactive), lightly active (moderately active/inactive), and active. ^3^ The original item of the index, “At least 1 serving of plant-based protein foods (legumes and nuts) in daily main meals (lunch and dinner)”, was adapted to “At least 2 servings of plant-based protein foods (legumes and nuts) per day” since the food frequency questionnaire (FFQ) does not capture meal timing. This ensures the intended daily intake is maintained. ^4^ Processed meat refers to meat that has been transformed through salting, curing, fermentation, smoking or other processes to enhance flavor or improve preservation.

**Table 2:** Baseline characteristics of AWHS participants according to healthy and sustainable dietary recommendations for Spanish population index quartiles.

		Q1 (1–5 pts)	Q2 (6 pts)	Q3 (7 pts)	Q4 (8–13 pts)	*p* for Trend
**N** ** = 2286**	**Mean**	**n** ** = 940**	**n** ** = 534**	**n** ** = 408**	**n** ** = 404**	
Age, y	51.6 (3.9)	51.4 (3.9)	51.6 (3.9)	51.8 (3.8)	51.9 (4.0)	0.007
Work shift, % (n)						<0.001
Central	8.8 (202)	7.9 (74)	8.2 (44)	10.3 (42)	10.4 (42)	
Morning/afternoon	60.0 (1372)	65.0 (611)	61.8 (330)	53.4 (218)	52.7 (213)	
Morning/afternoon/night	20.3 (465)	15.5 (146)	19.1 (102)	26.0 (106)	27.5 (111)	
Night	10.8 (247)	11.6 (109)	10.9 (58)	10.3 (42)	9.4 (38)	
Physical activity, total METs-h/wk	31.9 (22.7)	30.7 (21.6)	31.5 (21.4)	31.6 (23.5)	35.6 (25.4)	0.001
Ever-smokers, %	76.9 (1757)	77.3 (727)	79.4 (424)	74.5 (304)	74.8 (302)	0.173
Alcohol intake, g/d	20.7 (19.4)	20.8 (19.8)	21.1 (19.6)	20.6 (18.4)	20.15 (19.6)	0.573
BMI, kg/m^2^	27.9 (3.5)	27.9 (3.6)	28.1 (3.5)	28.1 (3.3)	27.8 (3.3)	0.993
Waist circumference, cm	98.2 (9.2)	98.3 (9.2)	98.6 (9.6)	98.2 (9.1)	97.2 (9.0)	0.094
Systolic blood pressure, mm Hg	125.7 (14.2)	125.2 (14.3)	126.2 (14.0)	126.4 (14.4)	125.6 (13.9)	0.369
Diastolic blood pressure, mm Hg	82.8 (9.5)	82.7 (9.7)	83.4 (9.1)	82.9 (9.6)	82.5 (9.4)	0.820
Total cholesterol, mg/dL	220.1 (36.7)	218.5 (36.7)	220.7 (35.8)	223.0 (34.9)	219.8 (39.6)	0.226
HDL-c, mg/dL	52.9 (11.3)	51.5 (11.1)	52.8 (10.7)	54.0 (11.7)	55.3 (11.8)	<0.001
LDL-c, mg/dL	137.1 (32.2)	136.1 (32.4)	138.3 (31.5)	139.1 (30.8)	136.0 (33.9)	0.622
Triglycerides, mg/dL	152.1 (99.7)	156.7 (101.1)	150.0 (99.7)	151.4 (98.5)	144.8 (97.3)	0.052
Fasting glucose, mg/dL	98.2 (17.9)	98.5 (21.0)	98.1 (15.3)	98.3 (15.5)	97.7 (15.4)	0.510
Hypertension, % (n)	38.8 (887)	36.3 (341)	37.3 (199)	43.1 (174)	42.3 (171)	0.008
Diabetes, % (n)	6.2 (141)	6.5 (61)	6.0 (32)	5.4 (22)	6.4 (26)	0.757
Dyslipidemia, % (n)	50.7 (1158)	47.3 (445)	51.1 (273)	55.6 (227)	52.7 (213)	0.012

Values are mean (SD) or % (number). *p* value for trend from unadjusted regression models. Abbreviations: BMI, body mass index; HDL-c, high-density lipoprotein cholesterol; LDL-c, low-density lipoprotein cholesterol; MET, metabolic equivalent; pts, points.

**Table 3:** Odds ratios (OR) (95% CI) for the association between healthy and sustainable dietary recommendations for Spanish population index and metabolic syndrome, as well as metabolic syndrome criteria in the AWHS participants.

	Per 1-SD Increase	Q1 (1–5 pts)	Q2 (6 pts)	Q3 (7 pts)	Q4 (8–13 pts)	*p* for Trend ^2^
	**n** ** = 2286**	**n** ** = 940**	**n** ** = 534**	**n** ** = 408**	**n** ** = 404**	
MetS, % (n)	27.6 (632)	27.7 (260)	29.4 (157)	28.9 (118)	24.0 (97)	
Adjusted for age	0.92 (0.84–1.01)	Ref.	1.07 (0.85–1.36)	1.03 (0.79–1.33)	0.78 (0.60–1.03)	0.083
Multivariable-adjusted ^1^	0.86 (0.77–0.97)	Ref.	0.93 (0.70–1.22)	0.90 (0.66–1.22)	0.72 (0.52–0.99)	0.013
Abdominal obesity, % (n)	32.4 (741)	32.6 (306)	35.4 (189)	31.4 (128)	29.2 (118)	
Adjusted for age	0.92 (0.84–1.01)	Ref.	1.12 (0.90–1.41)	0.92 (0.72–1.19)	0.83 (0.64–1.07)	0.067
Multivariable-adjusted ^1^	0.80 (0.70–0.92)	Ref.	0.94 (0.69–1.28)	0.67 (0.47–0.95)	0.69 (0.48–0.99)	0.001
Elevated fasting glucose, % (n)	38.2 (873)	36.6 (344)	39.9 (213)	40.0 (163)	37.9 (153)	
Adjusted for age	1.02 (0.94–1.11)	Ref.	1.13 (0.91–1.41)	1.11 (0.86–1.42)	1.01 (0.79–1.29)	0.655
Multivariable-adjusted ^1^	0.98 (0.89–1.08)	Ref.	1.04 (0.82–1.32)	1.01 (0.78–1.32)	0.95 (0.73–1.26)	0.662
High blood pressure, % (n)	57.0 (1304)	55.0 (517)	56.6 (302)	60.5 (247)	58.9 (238)	
Adjusted for age	1.05 (0.97–1.15)	Ref.	1.04 (0.84–1.30)	1.21 (0.97–1.54)	1.11 (0.88–1.42)	0.200
Multivariable-adjusted ^1^	1.03 (0.94–1.14)	Ref.	0.94 (0.75–1.19)	1.12 (0.87–1.45)	1.08 (0.83–1.41)	0.484
High triglycerides, % (n)	38.0 (868)	39.5 (371)	38.6 (206)	36.8 (150)	34.9 (141)	
Adjusted for age	0.90 (0.83–0.98)	Ref.	0.96 (0.77–1.19)	0.88 (0.69–1.13)	0.81 (0.64–1.04)	0.017
Multivariable-adjusted ^1^	0.91 (0.83–1.01)	Ref.	0.93 (0.74–1.17)	0.88 (0.68–1.14)	0.86 (0.66–1.12)	0.065
Low HDL-cholesterol, % (n)	9.4 (216)	10.2 (96)	9.9 (53)	8.3 (34)	8.2 (33)	
Adjusted for age	0.87 (0.75–1.00)	Ref.	0.97 (0.68–1.38)	0.80 (0.53–1.20)	0.78 (0.52–1.18)	0.058
Multivariable-adjusted ^1^	0.88 (0.75–1.03)	Ref.	0.97 (0.67–1.40)	0.83 (0.54–1.27)	0.83 (0.54–1.30)	0.122

Abbreviations: HDL, high-density lipoprotein; MetS, metabolic syndrome; N, total number of participants; OR, odds ratio; Ref., reference; pts, points. ^1^ Logistic regression models adjusted for age, shift work (central, morning/afternoon, morning/afternoon/night, and night), body mass index (<25, 25–29.9, and ≥30 kg/m^2^), smoking status (ever smoker or never smoker), physical activity (total metabolic equivalents-h/wk), total energy (kcal/d), alcohol consumption (g/d). ^2^ *p* for trend is calculated using healthy and sustainable dietary recommendations for Spanish index as a continuous variable. 1-SD = 1.787402.

## Data Availability

The original contributions presented in this study are included in the article. Further inquiries can be directed to the corresponding author.

## Supplementary Materials

The following supporting information can be downloaded at: https://www.mdpi.com/article/10.3390/nu17233725/s1, Table S1. Distribution of participants in the healthy and sustainable dietary recommendations for Spanish population index; Table S2. Adherence of AWHS participants to the healthy and sustainable dietary recommendations for the components of the Spanish population index (N = 2286); Table S3. Nutritional Status and Food Intake of AWHS participants according to healthy and sustainable dietary recommendations for Spanish populations index quartiles; Table S4. Environmental impact measured by greenhouse gas emissions (GHGE in kg CO_2_-eq/day) from all AWHS participants’ diets; Figure S1. Odds ratios (OR) (95% CI) for metabolic syndrome and its criteria per 1 standard deviation increase in the healthy and sustainable dietary recommendations for Spanish populations index.

## Abbreviations

The following abbreviations are used in this manuscript:

AESAN	Spanish Agency for Food Safety and Nutrition
AWHS	Aragon Workers Health Study
BMI	Body Mass Index
CEICA	Clinical Research Ethics Committee of Aragon
CIs	Confidence Intervals
CVD	Cardiovascular Disease
DASH	Dietary Approaches to Stop Hypertension
FFQ	Food Frequency Questionnaire
GHGE	Greenhouse Gas Emissions
HDL-c	High-Density Lipoprotein Cholesterol
HS-DRSI	Healthy and Sustainable Dietary Recommendations for the Spanish Population Index
MetS	Metabolic Syndrome
NCEP-ATP III	National Cholesterol Education Programme-Adult Treatment Panel III
SD	Standard Deviation
ORs	Odds ratios
PHD	Planetary Health Diet
