# Supplementary material for: Metabolic and Environmental Benefits of Following the Healthy and Sustainable Dietary Recommendations for the Spanish Population: The AWHS Study
Source: Nutrients. 2025 Nov 27;17(23):3725. doi: 10.3390/nu17233725 (PMC12694131; doi:10.3390/nu17233725)
Supplement: Supplementary file 1 [file nutrients-17-03725-s001.zip › nutrients-3972201-supplementary.pdf]

nutrients MDPI

Article

Metabolic and Environmental Benefits of Following the Healthy and Sustainable Dietary Recommendations for the Spanish Population: The AWHS Study

Sofía Gimeno-Ruiz [1], Pilar Guallar-Castillón [2,3,4,*], Esther López-García [2,3], Carolina Torrijo-Belanche [1], Ainara Muñoz-Cabrejas [5], María Morales-Suárez-Varela [6,7] and Belén Moreno-Franco [1,5,8]

[1] Department of Preventive Medicine and Public Health, Universidad de Zaragoza, 50009 Zaragoza, Spain; gimenoruizsofia@gmail.com (S.G.-R.); carolinatorrijob@gmail.com (C.T.-B.); mbmoreno@unizar.es (B.M.-F.)
[2] Instituto Madrileño de Estudios Avanzados en Alimentación (IMDEA-Food Institute), Campus de Excelencia Internacional Universidad Autónoma de Madrid + Consejo Superior de Investigaciones Científicas (CEI UAM + CSIC), 28049 Madrid, Spain; esther.lopez@uam.es
[3] Department of Preventive Medicine and Public Health, School of Medicine, Universidad Autónoma de Madrid, 28029 Madrid, Spain
[4] Department of Epidemiology, New York University School of Global Public Health, 10003 New York, United State of America
[5] Instituto de Investigación Sanitaria Aragón, Hospital Universitario Miguel Servet, 50009 Zaragoza, Spain; ainaramunozc@gmail.com
[6] Department of Preventive Medicine and Public Health, Food Sciences, Toxicology and Forensic Medicine, Universitat de València, 46100 Valencia, Spain; maria.m.morales@uv.es
[7] Centro de Investigación Biomédica en Red de Epidemiología y Salud Pública (CIBERESP), 28029 Madrid, Spain
[8] Centro de Investigación Biomédica en Red de Enfermedades Cardiovasculares (CIBERCV), 28029 Madrid, Spain
* Correspondence: mpilar.guallar@uam.es

Academic Editor: Caroline S. Stokes

Received: 22 October 2025
Revised: 24 November 2025
Accepted: 26 November 2025
Published: 27 November 2025

Citation: Gimeno-Ruiz, S.; Guallar-Castillón, P.; López-García, E.; Torrijo-Belanche, C.; Muñoz-Cabrejas, A.; Morales-Suárez-Varela, M.; Moreno-Franco, B. Metabolic and Environmental Benefits of Following the Healthy and Sustainable Dietary Recommendations for the Spanish Population: The AWHS Study. *Nutrients* **2025**, *17*, 3725. https://doi.org/10.3390/nu17233725

Copyright: © 2025 by the authors. Submitted for possible open access publication under the terms and conditions of the Creative Commons Attribution (CC BY) license (https://creativecommons.org/licenses/by/4.0/).

Supplementary Material **Table S1.** Distribution of participants in the healthy and sustainable dietary recommendations for Spanish population index.

Index Score	Frequency	Percentage	Cumulative
1	4	0.17	0.17
2	40	1.17	1.92
3	113	4.94	6.87
4	302	13.21	20.08
5	481	21.04	41.12
6	534	23.36	64.48
7	408	17.85	82.33
8	210	9.19	91.51
9	111	4.86	96.37
10	58	2.54	98.91
11	21	0.92	99.83
12	3	0.13	99.96
Total	2286	100.00	

Table S2. Adherence of AWHS participants to the healthy and sustainable dietary recommendations for the components of the Spanish population index (N = 2286).

	Index Components	% Compliance (N)
1	Vegetables and fruits, excluding fruit juice	20.8 (475)
2	At least 3 servings of V&F are vegetables	8.0 (182)
3	Potatoes and other tubers	63.8 (1459)
4	Cereals, depending on the level of energy requirement	74.4 (1701)
5	At least half of the servings/d of cereals are whole grain products	10.5 (240)
6	Legumes	0.4 (8)
7	Nuts	33.4 (764)
8	At least 2 servings of plant-based protein foods (nuts and legumes) per day	2.2 (50)
9	Fish and seafood	70.0 (1601)
10	At least half of the servings of fish and seafood are oily fish	13.0 (296)
11	Eggs	87.0 (1988)
12	Dairy	85.9 (1963)
13	All types of meat	0.7 (15)
14	At least half of the servings of meat are white meat from poultry or rabbit	10.2 (233)
15	Olive oil for cooking and food dressing	40.2 (920)
16	Minimize consumption of processed meat	0.5 (12)
17	Minimize consumption of other processed foods high in sugars, fats, and salt (e.g., industrially baked foods, cookies, chocolate, sweets, snacks)	0.8 (18)
18	Minimize consumption of salt	46.2 (1056)
19	Minimize consumption of sweetened beverages (sugar-sweetened, artificially sweetened, fruit juices) and energy drinks	28.6 (654)
20	Water as the drink of choice	-

Table S3. Nutritional Status and Food Intake of AWHS participants according to healthy and sustainable dietary recommendations for Spanish population index quartiles.

		Q1	Q2	Q3	Q4	
**N = 2286**	**Mean**	**n = 940**	**n = 534**	**n = 408**	**n = 404**	***p* for Trend**
Macronutrients						
Energy, kcal/d	2812.4 (614.2)	3033.7 (586.0)	2725.1 (586.0)	2652.0 (589.9)	2574.6 (580.2)	<0.001
Carbohydrates, g/d	318.9 (93.7)	359.6 (90.3)	301.4 (85.4)	292.1 (86.6)	274.6 (81.4)	<0.001
Protein, g/d	105.7 (22.8)	109.7 (22.0)	103.6 (23.3)	102.2 (22.3)	102.2 (23.3)	<0.001
Fat, g/d	107.7 (25.1)	112.4 (23.9)	106.4 (25.7)	103.4 (24.9)	102.9 (25.6)	<0.001
Fiber, g/d	24.9 (7.6)	23.9 (6.5)	23.6 (6.9)	24.9 (7.6)	28.9 (9.2)	<0.001
Vegetal origin food						
Vegetables, g/d	332.1 (135.0)	297.9 (109.1)	319.1 (117.4)	350.7 (143.4)	410.4 (164.6)	<0.001
Fruit, g/d	321.7 (178.7)	274.5 (150.8)	321.5 (171.0)	348.1 (197.3)	404.9 (192.9)	<0.001
Potatoes and other tubers, g/d	92.9 (45.9)	101.5 (45.6)	89.5 (43.4)	88.5 (46.6)	81.9 (45.9)	<0.001
Total cereals, g/d	211.1 (117.8)	262.0 (123.2)	188.7 (105.7)	176.9 (99.2)	156.9 (88.2)	<0.001
Whole grain cereal, g/d	19.6 (54.6)	10.4 (45.7)	16.8 (48.4)	21.0 (53.5)	43.2 (72.7)	<0.001
Legumes, g/d	40.1 (18.2)	39.6 (13.3)	40.4 (21.5)	39.7 (16.1)	41.1 (24.2)	0.271
Nuts, g/d	10.2 (12.2)	6.3 (8.2)	8.8 (10.1)	11.9 (12.6)	19.2 (16.2)	<0.001
Olive oil, g/d	32.8 (14.3)	32.0 (14.4)	32.3 (14.8)	34.5 (14.4)	33.5 (13.1)	0.009
Animal-origin food						
Total fish and seafood, g/d	85.7 (43.6)	73.7 (39.9)	86.3 (45.2)	95.1 (42.6)	103.5 (42.5)	<0.001
Oily fish, g/d	25.7 (19.5)	20.3 (15.6)	25.3 (18.4)	29.3 (20.7)	35.0 (23.0)	<0.001
Eggs, g/d	25.7 (15.3)	29.0 (16.6)	24.8 (14.2)	22.4 (10.8)	22.9 (16.0)	<0.001
Dairy and dairy products, g/d	303.2 (202.7)	321.5 (222.3)	290.4 (191.2)	308.3 (190.4)	272.4 (175.4)	<0.001
Total meat and meat products, g/d	183.0 (67.0)	190.5 (64.0)	186.9 (68.5)	174.5 (64.1)	168.8 (71.7)	<0.001
Poultry or rabbit meat, g/d	57.6 (31.2)	54.1 (28.5)	59.0 (29.9)	57.3 (29.9)	64.0 (38.3)	<0.001
Processed food and drinks						
Processed meat, g/d	36.4 (18.9)	39.2 (18.1)	37.2 (19.9)	33.6 (17.5)	31.4 (19.4)	<0.001
Processed foods high in sugars, fats, and salt, g/d	101.2 (63.3)	111.2 (70.1)	99.3 (58.0)	92.5 (57.1)	89.4 (55.7)	<0.001
Sweetened beverages and energy drinks, g/d	134.5 (165.6)	171.8 (165.6)	125.3 (149.3)	107.8 (146.8)	86.8 (130.7)	<0.001
Others						
Salt, g/d	2.7 (1.4)	3.0 (1.4)	2.7 (1.4)	2.4 (1.4)	2.1 (1.3)	<0.001

Values are expressed as means (SE). *p* value for trend from unadjusted regression model.

Table S4. Environmental impact measured by greenhouse gas emissions (GHGE in kg CO_2-eq/day) from all AWHS participants' diets.

Food Categories	Intake (kg/day)	GHGE (kg CO_2-eq/day)	Percentage
Vegetables	0.33 (0.13)	0.36 (0.15)	5.96
Fruits	0.32 (0.18)	0.20 (0.12)	3.41
Potatoes and other tubers	0.09 (0.05)	0.08 (0.04)	1.28
Cereals and processed cereal products	0.21 (0.12)	0.22 (0.12)	3.63
Legumes	0.04 (0.02)	0.04 (0.02)	0.74
Nuts and seeds	0.01 (0.01)	0.02 (0.02)	0.29
Vegetable oils	0.05 (0.02)	0.13 (0.05)	2.22
Butter and animal fats	<0.01	0.01 (0.02)	0.09
Fish and seafood	0.09 (0.04)	0.43 (0.22)	7.16
Eggs	0.03 (0.02)	0.07 (0.04)	1.25
Dairy and dairy-based products (e.g., milkshakes or ice cream)	0.33 (0.21)	0.67 (0.35)	11.17
Red meat and processed meats	0.12 (0.05)	2.57 (1.32)	43.06
Poultry and rabbit meat	0.06 (0.03)	0.47 (0.30)	7.89
Ultra-processed foods high in sugar, fat, and/or salt (e.g., snacks, pastries, sweets, sauces, honey)	0.11 (0.05)	0.18 (0.11)	3.06
Sugar-sweetened beverages, energy drinks, alcoholic drinks, coffee, tea, etc.	0.54 (0.43)	0.52 (0.44)	8.78
Total		5.97 (1.62)	100.00

Values are mean (SD). Abbreviations: GHGE: greenhouse gas emissions.

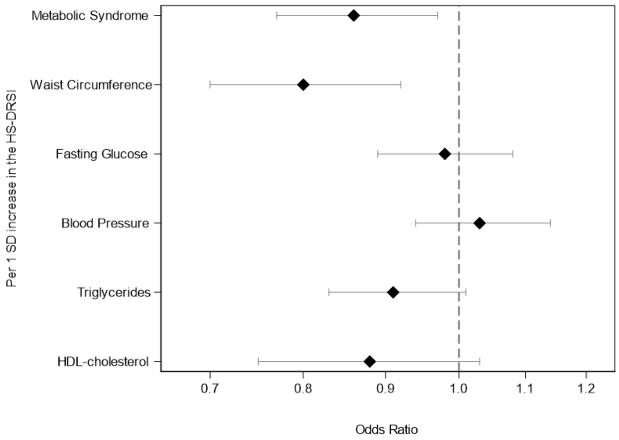

Figure S1. Odds ratios (OR) (95% CI) for metabolic syndrome and its criteria per 1 standard deviation increase in the healthy and sustainable dietary recommendations for Spanish population index. The diamond symbols show the ORs, with the horizontal lines representing the 95% CIs.
